# American Armamentarium Chirurgicum; Tieman

**Published:** 1889-10

**Authors:** 


AMERICAN ARMAMENTARIUM CHIRURGICUM. Geo. Tieman & Co., New York.

A handsome quarto volume of 846 pages, in cloth and morocco, gilt, and illustrated with a cut of every instrument and surgical appliance, (and their names is legion), and part of instrument, known to modern surgery, together with cuts of the numerous medals which have, from time to time, been bestowed on Tieman's instruments for excellence over all others, in various parts of the word. Tieman is the manufacturer par excellence.

Most persons regard such publications as this, as mere catalogues of the manufacturers' wares, sent out solely for advertising purposes. While it is a splendid advertisement, and in this instance, an expensive one, yet such a work as the one before us fills a larger measure of usefulness than a mere catalogue, and is a real help to the surgeon, physician and student, and therefore, is worthy of a place in the library; for, as the publisher says in the preface:

"In surgical works many of the instruments for performing operations are not illustrated. In illustrated catalogues, on the other hand, a description of the modus operandi is wanting.' Thus it will be seen this work is useful as an adjuvant or supplement to the surgical text books; the catalogue gives a clearer idea of the instrument required and appliances for deformity and injury than any words can convey. And in this work not only are the instrument and appliances figured and described, but their application is illustrated also; and even more, many useful hints and suggestions are given—when it comes to a question of what operation to perform; with an outline of the views of eminent modern authority on surgical diseases and injuries of nearly every kind. It is a most convenient and useful book of ready reference, and an ornament besides.

